# Analysis of DNA variations in *GSTA* and *GSTM* gene clusters based on the results of genome-wide data from three Russian populations taken as an example

**DOI:** 10.1186/1471-2156-13-89

**Published:** 2012-10-22

**Authors:** Irina N Filippova, Andrey V Khrunin, Svetlana A Limborska

**Affiliations:** 1 Department of Molecular Bases of Human Genetics, Institute of Molecular Genetics, Russian Academy of Sciences, Moscow, Russia

**Keywords:** Single nucleotide polymorphism, *GSTM*, *GSTA*, Haplotypes

## Abstract

**Background:**

Extensive genome-wide analyses of many human populations, using microarrays containing hundreds of thousands of single-nucleotide polymorphisms, have provided us with abundant information about global genomic diversity. However, these data can also be used to analyze local variability in individual genomic regions. In this study, we analyzed the variability in two genomic regions carrying the genes of the *GSTA* and GSTM subfamilies, located on different chromosomes.

**Results:**

Analysis of the polymorphisms in *GSTA* and *GSTM* gene clusters showed similarities in their allelic and haplotype diversities. These patterns were similar in three Russian populations and the CEU population of European origin. There were statistically significant differences in all the haploblocks of both the *GSTM* and *GSTA* regions when the Russian populations were compared with populations from China and Japan. Most haploblocks also differed between the Russians and Nigerians from Yoruba, but, some of them had similar allelic frequencies. Special attention was paid to SNP rs4986947 from the intron of the *GSTA4* gene, which is represented in apes by an A nucleotide. In the Asian and African samples, it was represented only by a G allele, and both allelic variants (G/A) occurred in the Russian and European populations.

**Conclusions:**

The results obtained suggest the presence of common features in the evolutionary histories of the *GSTA* and *GSTM* gene regions, and that African subpopulations were involved differently in the formation of the European and Asian human lineages.

## Background

The results of genome-wide analyses of different populations can be used to study the patterns of DNA diversity in particular genomic regions containing specific genes or gene clusters. One interesting and functionally significant genetic system includes the glutathione-S-transferase (GST) genes that encode the different GSTs.

The GSTs are one of the key groups of detoxification enzymes. The chemistry of the reactions catalyzed by these enzymes is based predominantly on the conjugation of glutathione to the electrophilic centers of various substances, which leads to a loss of toxicity and the formation of more hydrophilic products. The important noncatalytic functions of the GSTs include their capacity to sequester carcinogens, their involvement in the intracellular transport of a wide spectrum of hydrophobic ligands, and their modulation of signaling pathways [1,2]. Like most other human genes, the genes encoding the GSTs are polymorphic. It has been suggested that these polymorphisms are functionally significant and that the frequencies of their allelic variants differ among human populations [3]. Until recently, only a limited number of *GST* polymorphisms had been studied (e.g., *GSTM1* and *GSTT1* gene deletions, a 3-bp deletion in *GSTM3* intron 6, and SNPs in *GSTP1* exons 5 and 6), and these were not sufficient to infer the genetic relationships of populations. It may be especially relevant that particular genes of some GST families are located close to each other, forming clusters in the genome [4]. However, with advances in the methods of genome analysis, including high-throughput genotyping technologies, it has become possible to obtain and use more detailed information about the polymorphisms in regions of interest. Recently, Polimanti and co-workers (2011) [5] compared polymorphisms of the soluble GST genes in some reference populations using the HapMap database. In the current study, we examined the polymorphisms in two genomic regions, comprising clusters of *GSTA* and *GSTM* genes, located on different chromosomes, in three groups of Russians from the western (Tver), eastern (Murom), and southern (Kursk) regions of the European part of Russia. The analyses were based on both the comparison of allelic variation in individual SNPs and the haplotype diversity across the GST clusters. The genotypes were obtained from a genome-wide analysis of SNPs [6,7], performed with Illumina microarrays. To compare the Russian populations with other populations throughout the world, we also included four populations from the HapMap Project in this study: Utah USA residents with ancestry in northern and Western Europe (СEU), Han Chinese from Beijing (CHB), Japanese from Tokyo (JPT), and the Yoruba people of Ibadan, Nigeria (YRI). Their genotypes were downloaded from the HapMap Project site [8]. The data obtained showed high levels of similarity across the three Russian populations studied and between the Russian and CEU populations. However, the differences between them and the Asian and African populations were significant.

## Methods

DNA samples were isolated from blood samples obtained with the informed consent of Russian donors from western (Andreapol district of the Tver region), eastern (Murom district of the Vladimir region), and southern locations (Kursk and Oktyabrsky districts of the Kursk region) in the European part of Russia. Their ethnicity was determined by interview. All individuals were unrelated and represented the native ethnic groups in the regions studied (i.e., they belonged to at least the third generation living in a particular geographic region). The DNA was isolated from peripheral leukocytes with standard techniques, using proteinase K treatment and phenol–chloroform extraction [9].

All the DNA samples were genotyped at the Estonian Biocentre (Tartu, Estonia), using the Illumina Human CNV370-Duo (Tver and Murom samples) and Human 660W-Quad chips (Kursk samples), according to the manufacturer’s instructions. In total, 288 Russian samples were genotyped (96 samples per population). Because the microarrays differed in the numbers of SNPs tested, the number of SNPs examined was standardized to obtain a set of loci that was consistent across all the populations analyzed. The set of loci was chosen by considering the chromosomal regions in which the *GSTM* and *GSTA* gene clusters are located. The sample sizes of the populations taken from the HapMap Project were: 165 individuals from CEU, 86 from CHB, 84 from JPT, and 166 from YRI.

The allele frequencies, their Hardy–Weinberg equilibrium status, and the SNP-based Wright’s fixation index (F_ST_) [10] were calculated using the PowerMarker software package (v.3.0) [11]. The pairwise linkage disequilibrium statistic (*D'*) [12] was estimated and the haplotypes were inferred for adjacent markers using an accelerated expectation-maximization algorithm embedded in the Haploview software [13]. The haplotype block patterns were defined using the block definition based on the linkage disequilibrium measure *D'* and its confidence interval. Linearized pairwise F_ST_[14] values were used to evaluate the genetic affinities between populations. The significance level was set at *P* < 0.05.

## Results

**Figure 1:**
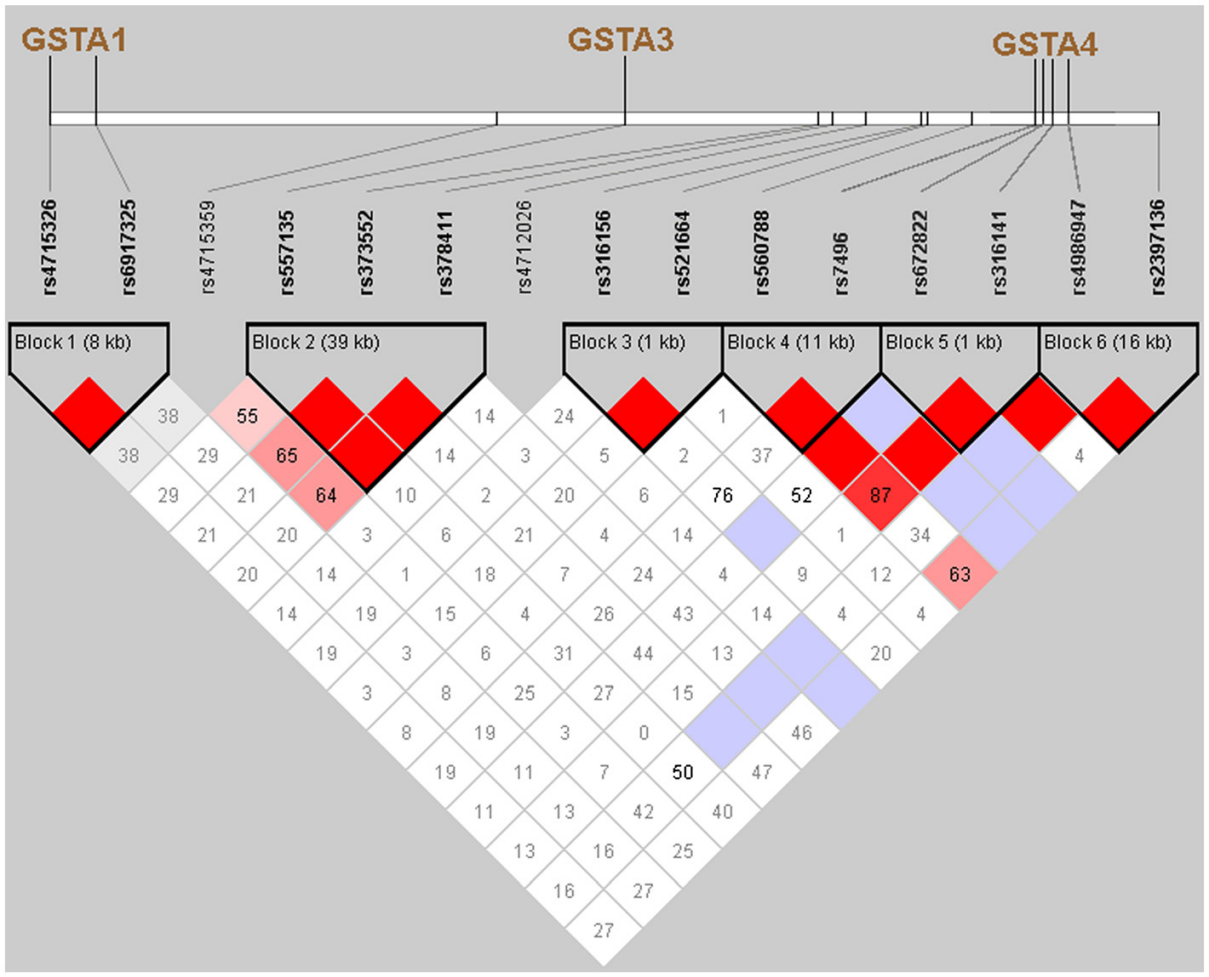
**SNPs studied in the GSTA gene cluster (e.g., the Tver population).** The numbers inside the diamonds show the pairwise linkage disequilibrium (*D*^′^) values.

Figure 1 shows 15 polymorphisms of the *GSTA* cluster, which is located at p12.1 of chromosome 6 over a 250-kbp area. The polymorphisms are presented according to their locations in relation to the genes. Based on the threshold value for the pairwise linkage disequilibrium between the SNPs (*D*' > 0.7) [15], six blocks were inferred in the *GSTA* cluster. All the haploblocks were identical in all the populations studied. Figure 1 shows the haploblocks for the Russian population from Tver. The corresponding data for the other two Russian populations were identical to the Tver data.

Table 1 shows the allelic frequencies for all the polymorphic loci of the *GSTA* cluster in the Russian populations and in the HapMap populations. A comparative analysis showed no differences in the distributions of the SNP variants in the Russian populations, and similar allelic frequencies were found in the CEU population. However, the allelic distributions in the three remaining HapMap populations differed considerably from those in the populations of European descent.

**Table 1:** **Minor allelic frequencies of polymorphisms in *GSTA *cluster**

**Marker**	**Allele**	**Tver**	**Murom**	**Kursk**	**CEU**	**CHB**	**JPT**	**YRI**
		**2N=192**	**2N=192**	**2N=192**	**2N=330**	**2N=168**	**2N=172**	**2N=332**
rs4715326	C	0.396	0.400	0.403	0.406	0.095	0.128	0.299
rs6917325	T	0.396	0.370	0.416	0.409	0.096	0.128	0.298
rs4715359	G	0.339	0.292	0.346	0.312	0.133	0.151	0.260
rs557135	G	0.318	0.313	0.321	0.348	0.500	0.477	0.689
rs373552	G	0.245	0.234	0.223	0.279	0.042	0.105	0.022
rs378411	G	0.240	0.234	0.223	0.279	0.042	0.105	0.048
rs4712026	G	0.141	0.094	0.121	0.128	0.337	0.244	0.111
rs316156	G	0.135	0.146	0.089	0.161	0.226	0.291	0.117
rs521664	C	0.396	0.375	0.432	0.288	0.559	0.547	0.078
rs560788	T	0.495	0.464	0.500	0.461	0.232	0.227	0.629
rs7496	T	0.172	0.172	0.132	0.1	0.149	0.088	0.247
rs672822	G	0.063	0.125	0.101	0.104	0	0	0.060
rs316141	A	0.443	0.438	0.458	0.445	0.253	0.250	0.482
rs4986947	A	0.078	0.052	0.104	0.055	0	0	0
rs2397136	G	0.151	0.125	0.161	0.145	0.048	0.111	0.299

**Figure 2:**
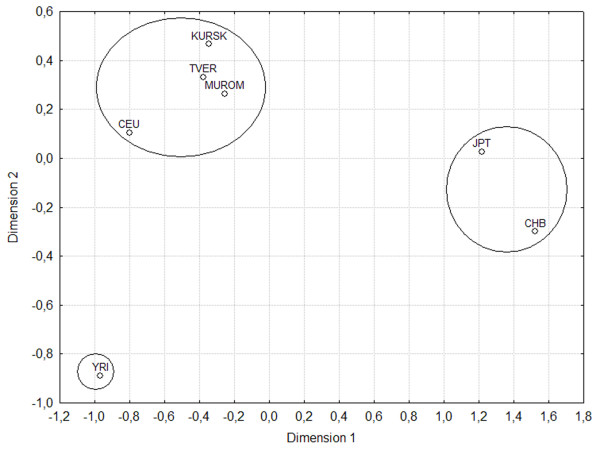
Two-dimensional scaling plot of the matrix of genetic distances between the Russian populations (Tver, Murom, and Kursk) and populations from the HapMap Project (CEU, CHB, JPT, and YRI).

We also calculated the fixation indices (F_ST_) to quantitatively assess the levels of interpopulation frequency variation. Figure 2 presents the multidimensional scaling of the matrix of linearized pairwise F_ST_ values. The diagram shows that the Russian populations form a single cluster, with the CEU population close to them. However, the African YRI population and Asian CHB and JPT populations are situated at a considerable distance from them.

Table 2 shows the frequencies of the haplotypes in each haploblock in all the populations analyzed. It is evident that different haploblocks contain different numbers of haplotypes. For instance, haploblock #1 has only two haplotypes, whereas haploblock #4 has four haplotypes. Some haploblocks, namely blocks #5 and #6 in the CHB and JPT populations and block #6 in the YRI population, were not inferred because the SNPs tested were monomorphic in these populations.

**Table 2:** Haplotype frequencies in six selected blocks inferred for the *GSTA *cluster*

**Number of haploblock**	**Haplotype**	**Tver**	**Murom**	**Kursk**	**CEU**	**CHB**	**JPT**	**YRI**
I	ТС	0.604	0.630	0.590	0.594	0.905	0.872	0.701
	CT	0.396	0.370	0.410	0.406	0.095	0.128	0.299
II	AAA	0.682	0.686	0,670	0.652	0.500	0.523	0.311
	GGG	0.245	0.234	0,217	0.279	0.042	0.105	0.021
	GAA	0.073	0.079	0,107	0.107	0.458	0.372	0.641
	GAG	0	0	0	0	0	0	0.027
III	TT	0.469	0.479	0.479	0.551	0.214	0.163	0.810
	TC	0.396	0.375	0.432	0.288	0.560	0.547	0.073
	GT	0.135	0.146	0.089	0.161	0.226	0.291	0.112
IV	CC	0.505	0.529	0.494	0.534	0.755	0.767	0.361
	TC	0.323	0.299	0.374	0.367	0.097	0.146	0.391
	TT	0.172	0.164	0.120	0.094	0.136	0.081	0.238
	СТ	0	0	0,011	0	0.013	0	0
V	AG	0.557	0.562	0.547	0.556	-	-	0.518
	AA	0.380	0.306	0.351	0.340	-	-	0.422
	GA	0.063	0.131	0.101	0.104	-	-	0.060
VI	GA	0.849	0.869	0.831	0.855	-	-	-
	AG	0.078	0.046	0.089	0.055	-	-	-
	GG	0.073	0.079	0.074	0.091	-	-	-

*Because some haploblocks are absent in some populations (CHB, JPT, and YRI), the corresponding lines in the columns are marked by dashes.

The comparison of the populations was based on the haplotype frequencies calculated for each block. The calculated probabilities (*P* values) presented in Table 3 show the results of this comparison. The statistically significant levels of *P* were set for each block using the Bonferroni correction for multiple testing. The data generated showed no marked differences in the haplotype frequencies across the Russian populations and the CEU population. However, a comparison of the haplotype frequencies in the Russian populations with those in the Chinese, Japanese, and Nigerian populations indicated significant difference between them. Most *P* values were considerably lower than the specified levels. The exceptions were block #5 and, to a certain degree, blocks #1 and #4, where the pairwise *P* values for the pairs of Russian and Nigerian populations were higher than values specified for these blocks.

**Table 3:** Comparison of the haplotype frequencies in the *GSTA *gene cluster of the populations from Tver, Murom, and Kursk with those from the HapMap Project (CEU, CHB, JPT, and YRI) *

	**MUROM**	**KURSK**	**СЕU**	**CHB**	**JPT**	**YRI**	**Number of haploblock**	**The level of significance**
TVER	0,6746	0,8353	0,8534	<0,0001	<0,0001	0,0269	I	0,004
	0,9599	0,4282	0,6984	<0,0001	<0,0001	<0,0001	II	0,0025
	0,9030	0,3306	0,0403	<0,0001	<0,0001	<0,0001	III	0,0025
	0,8238	0,2076	0,0326	<0,0001	<0,0001	0,0065	IV	0,0019
	0,0484	0,3957	0,2428	**―**	**―**	0,6452	V	0,0025
	0,4538	0,9276	0,4634	**―**	**―**	―	VI	0,0025
MUROM		0,4641	0,4574	<0,0001	<0,0001	<0,0001	I	0,004
		0,5661	0,5307	<0.0001	<0.0001	<0,0001	II	0,0025
		0,1764	0,1188	<0.0001	<0.0001	<0,0001	III	0,0025
		0,1599	0,0408	<0,0001	<0,0001	0,0006	IV	0,0019
		0,5052	0,5524	**―**	**―**	0,0031	V	0,0025
		0,2664	0,8195	**―**	**―**	―	VI	0,0025
KURSK			0,9266	<0,0001	<0,0001	0,0097	I	0,004
			0,1321	<0.0001	<0.0001	<0,0001	II	0,0025
			0,0013	<0.0001	<0.0001	<0.0001	III	0,0025
			0,2053	<0,0001	<0,0001	0,0006	IV	0,0019
			0,9682	**―**	**―**	0,1181	V	0,0025
			0,2694	**―**	**―**	**―**	VI	0,0025

* Pairwise P-values are presented. The last column contains Bonferroni adjusted P-values calculated for each block. The calculations was performed using the formula *P* = 0.05/ ([n × a] – 1), where “n” is the number of haplotypes and “a” is the number of populations [16]. The absence of some haploblocks in the CHB, JPT, and YRI populations did not allow us to compare these populations; the respective columns are marked with dashes on the corresponding lines.

**Figure 3:**
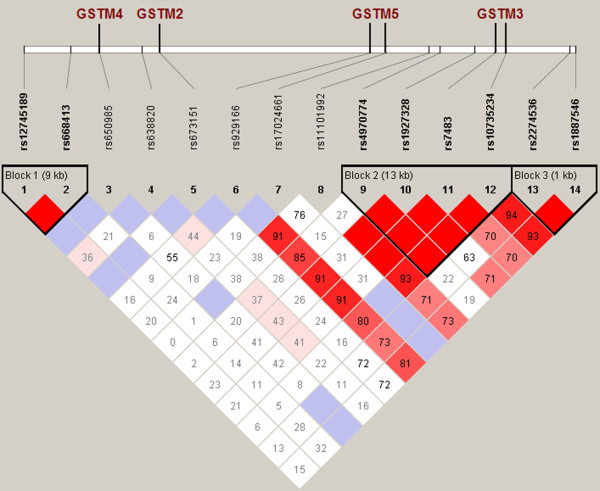
**SNPs studied in the *GSTM *gene cluster (e.g., the Tver population).**

The *GSTM* gene cluster is located on chromosome 1 in the p13.3 region and accounts for 85 kbp. The 14 marker loci found within the cluster (Figure 3) are listed in Table 4. As in the *GSTA* cluster, similarities in the frequencies of the *GSTM* alleles were observed between the Russian populations and the CEU population. Different frequencies were observed for the samples from Asia (CHB and JPT) and Africa (YRI). The two-dimensional plot of F_ST_-based distances was similar to the plot obtained for the *GSTA* cluster (data for the *GSTM* cluster are not shown).

**Table 4:** **Minor allelic frequencies of the polymorphisms in the *GSTM *cluster**

**Marker**	**Allele**	**Tver**	**Murom**	**Kursk**	**CEU**	**CHB**	**JPT**	**YRI**
		**2N=192**	**2N=192**	**2N=192**	**2N=330**	**2N=168**	**2N=172**	**2N=332**
rs12745189	T	0,443	0,458	0,495	0,402	0,613	0,686	0,377
rs668413	A	0,458	0,411	0,400	0,463	0,317	0,291	0,467
rs650985	C	0,042	0,063	0,054	0,070	0	0	0,003
rs638820	G	0,432	0,474	0,463	0,479	0,369	0,318	0,452
rs673151	T	0,036	0,063	0,053	0,042	0,012	0	0,015
rs929166	G	0,281	0,333	0,289	0,258	0,720	0,622	0,120
rs17024661	G	0,021	0,037	0,011	0,042	0,006	0	0,039
rs11101992	C	0,307	0,266	0,307	0,250	0,133	0,140	0,809
rs4970774	C	0,411	0,458	0,414	0,439	0,732	0,674	0,521
rs1927328	G	0,279	0,349	0,287	0,297	0,756	0,721	0,036
rs7483	T	0,266	0,339	0,287	0,299	0,756	0,721	0,033
rs10735234	G	0,391	0,375	0,379	0,430	0,143	0,180	0,096
rs2274536	G	0,307	0,313	0,344	0,288	0,113	0,151	0,632
rs1887546	T	0,297	0,313	0,339	0,288	0,095	0,112	0,596

Table 5 shows the haplotype frequencies in the haploblocks of the *GSTM* cluster. As in the *GSTA* cluster, the numbers of haplotypes observed in the blocks differed. When we considered the *P* values for the pairwise comparisons (Table 6), there were no marked differences in the haplotype frequencies between the Russian populations from Tver, Murom, and Kursk, and the CEU population. However, statistically significant differences were observed in most comparisons of the Russian populations with the CHB, JPT, and YRI populations. The only exceptions were in block #1, where the *P* values for the pairwise comparisons of the haplotype frequencies of the Russian and Nigerian populations were much higher than the specified significance level.

**Table 5:** **Haplotype frequencies in the three haploblocks of the *GSTM *cluster**

**Number of haploblock**	**Haplotypes**	**Tver**	**Murom**	**Kursk**	**CEU**	**CHB**	**JPT**	**YRI**
I	TC	0.443	0.458	0.495	0.395	0,628	0,654	0,451
	CA	0.458	0.411	0.400	0.456	0,314	0,297	0,414
	CC	0.099	0.130	0.105	0.142	0,058	0,036	0,136
	ТА	0	0	0	0	0	0,012	0
II	AACG	0.391	0.365	0.378	0.430	0.150	0.179	0.092
	CGTA	0.266	0.339	0.287	0.297	0.743	0.690	0.035
	AACA	0.198	0.167	0.213	0.130	0.083	0.071	0.377
	CACA	0.141	0.120	0.122	0.142	0	0	0.494
	CGTG	0	0	0	0	0.013	0	0
	AATA	0	0	0	0	0.012	0	0
	AGTA	0	0	0	0	0	0.060	0
III	AG	0.693	0.687	0.658	0.712	0.895	0.881	0.364
	GT	0.302	0.313	0.337	0.288	0.105	0.095	0.601
	GG	0	0	0	0	0	0.024	0.035

**Table 6:** Comparisons of the haplotype diversity of *GSTM *cluster in populations of Tver, Murom, Kursk and HapMap populations (CEU, CHB, JPT, YRI) *

	**MUROM**	**KURSK**	**СЕU**	**CHB**	**JPT**	**YRI**	**Number of haploblock**	**The level of significance**
TVER	0,5078	0,5183	0,2862	0,0021	<0,0001	0,4485	I	0,0025
	0,3202	0,8943	0,2160	<0,0001	<0,0001	<0,0001	II	0,0019
	0,9120	0,9206	0,7647	<0,0001	<0,0001	<0,0001	III	0,0025
MUROM		0,6541	0,3994	0,0027	<0,0001	0,5856	I	0,0025
		0,4052	0,1369	<0,0001	<0,0001	<0,0001	II	0,0019
		0,9392	0,5527	<0,0001	<0,0001	<0,0001	III	0,0025
KURSK			0,0811	0,0313	0,0018	0,1701	I	0,0025
			0,0906	<0,0001	<0,0001	<0,0001	II	0,0019
			0,8592	<0,0001	<0,0001	<0,0001	III	0,0025

* Pairwise P-values are presented. The last column contains Bonferroni adjusted P-values calculated for each block. The calculations was performed using the formula *P* = 0.05/ ([n × a] – 1), where “n” is the number of haplotypes and “a” is the number of populations [16].

## Discussion

Extensive genome-wide analyses of many human populations, using microarrays containing hundreds of thousands of SNPs, have provided us with considerable information about global genomic diversity [17]. These data can also be used to analyze the variability in local genomic regions, marking the evolutionary trajectories for both the main human groups and local populations.

In this study, we analyzed the variability of two genomic regions containing the genes of the *GSTA* and *GSTM* subfamilies. Our work was based on genotype data obtained from a whole-genome analysis of SNP genotypes performed with Illumina microarrays in three Russian populations. We compared these data with corresponding data from several HapMap populations.

Although genes of *GSTA* and *GSTM* subfamilies are located on different chromosomes, our analysis of the polymorphisms in these two gene clusters showed similarities between them in terms of their patterns of allelic and haplotype frequencies across the populations examined. The haplotype spectra of the three Russian populations studied (from Tver, Murom, and Kursk), who share a common ethnic origin, were similar. No marked differences were also established between the three Russian populations and the CEU population, which clearly reflects their common European ancestry. In this context, it was interesting to find some similarity between the Russian samples and the Yoruba population from Nigeria in the haplotype frequencies of some blocks (mainly block #5 of the *GSTA* cluster and block #1 of the *GSTM* cluster). Because the European populations differed significantly from the populations of China and Japan in the haplotype spectra of all blocks in both clusters, we propose that these similarities can be attributable to some particular features of these haploblocks in the microevolutionary history of the populations. At the same time, the Russian and Nigerian populations differed significantly in the remaining haploblocks of both gene clusters.

Another interesting finding that warrants particular attention is SNP rs4986947 from block #6 of the *GSTA* cluster, located in the intron of the *GSTA4* gene. In apes, this SNP site carries an A nucleotide [18]. In the populations analyzed from Asia and Africa, another nucleotide (G) occurred at this SNP site with a frequency of 100% (the same is also true for two other African HapMap samples—Luhya and Maasai) [19]. By contrast, in the European populations tested, including all populations from Russia, both alleles (G/A) are represented at this locus; i.e., the ancestral allele, containing A, is also present in these populations. Two possible explanations for this fact can be proposed. The first assumes substantial ancient gene flow (migrations) from Africa to the proto-West Eurasian (European) population after its divergence from the proto-East Eurasians [20]. These migrations could have included individuals with the ancestral A allele at rs4986947, which is virtually absent from the reference African populations. The second explanation is that the mutation could have been reversed in part of the European population, thus returning to its ancestral state. The persistence of the A allele in Europeans may be attributable to natural selection, which can shape the interethnic variation in the GST genes, as has been demonstrated by Polimanti et al. (2011) [5]. In addition to the Russian and CEU samples tested, the A allele at rs4986947 is also found at frequencies of around 6% in geographically distant European samples from Great Britain, Finland, and Italy [19]. These quite low frequencies may be the result of balancing selection.

## Conclusion

In summary, we have reported the results of a study of SNPs in two genomic regions carrying the genes of the *GSTA* and *GSTM* subfamilies. By using a haplotype-based approach, we have demonstrated a similarity in the patterns of allelic diversity between the *GSTA* and *GSTM* gene clusters in all populations studied. This leads us to propose that the evolutionary histories of these clusters share many features and mark the same events in the evolutionary trajectories of the main human groups.

## Competing interests

The authors declare no competing interests.

## Authors’ contributions

INF carried out the polymorphism typing, performed the statistical analysis and drafted the manuscript. AVK participated in the study design, helped with the statistical analysis and manuscript drafting. SAL conceived of the study, participated in its coordination and helped to draft the manuscript. All authors read and approved the final manuscript.
